# Reductions in expression of growth regulating genes in skeletal muscle with age in wild type and myostatin null mice

**DOI:** 10.1186/1472-6793-14-3

**Published:** 2014-03-28

**Authors:** Jennifer C Jones, Kellie A Kroscher, Anna C Dilger

**Affiliations:** 1 Department of Animal Sciences, University of Illinois at Urbana-Champaign, Urbana, IL 61801, USA

**Keywords:** *Ezh2*, *Gpc3*, *Mdk*, *Mest*, *Mycn*, *Peg3*, *Plagl1*, Muscle growth, Myostatin

## Abstract

**Background:**

Genes that decline in expression with age and are thought to coordinate growth cessation have been identified in various organs, but their expression in skeletal muscle is unknown. Therefore, our objective was to determine expression of these genes (*Ezh2*, *Gpc3*, *Mdk*, *Mest*, *Mycn*, *Peg3*, and *Plagl1*) in skeletal muscle from birth to maturity. We hypothesized that expression of these genes would decline with age in skeletal muscle but differ between sexes and between wild type and myostatin null mice.

**Results:**

Female and male wild type and myostatin null mice (C57BL/6J background) were sacrificed by carbon dioxide asphyxiation followed by decapitation at d -7, 0, 21, 42, and 70 days of age. Whole bodies at d -7, all muscles from both hind limbs at d 0, and bicep femoris muscle from d 21, 42 and 70 were collected. Gene expression was determined by quantitative real-time PCR. In general, expression of these growth-regulating genes was reduced at d 21 compared with day 0 and d -7. Expression of *Gpc3*, *Mest*, and *Peg3* was further reduced at d 42 and 70 compared with d 21, however the expression of *Mycn* increased from d 21 to d 42 and 70. Myostatin null mice, as expected, were heavier with increased biceps femoris weight at d 70. However, with respect to sex and genotype, there were few differences in expression. Expression of *Ezh2* was increased at d 70 and expression of *Mdk* was increased at d 21 in myostatin null mice compared with wild type, but no other genotype effects were present. Expression of *Mdk* was increased in females compared to males at d 70, but no other sex effects were present.

**Conclusions:**

Overall, these data suggest the downregulation of these growth-regulating genes with age might play a role in the coordinated cessation of muscle growth similar to organ growth but likely have a limited role in the differences between sexes or genotypes.

## Background

As humans and animals age, growth slows and eventually stops. Organs grow until they reach mature size; however, little is known about how growth cessation is regulated and coordinated in the body. A set of growth regulating genes, which include *Ezh2*, *Gpc3*, *Mdk*, *Mest*, *Mycn*, *Peg3*, and *Plagl1*, has been identified that is dependent upon growth and whose expression declines with age in organs [1,2]. In general, when expression of these genes decreases, proliferation decreases leading to a reduction in growth. Mutations of these genes also result in reduced viability, growth abnormalities, and diseases such as rhabdomyosarcoma, a skeletal muscle cancer, and Simpson-Golabi-Behmel syndrome [3-18].

Though well characterized in organs, these genes have not been well examined in growing muscle. It is expected that expression of these genes would differ between muscle, heart, and liver because of the differences in how these tissues grow. Postnatally, muscle grows by mainly hypertrophy, an increase in cell size, and not hyperplasia, an increase in cell number [19]. In mice, muscle fiber number becomes fixed at approximately d 7 postnatal [20]. Hypertrophy of muscle fibers is accompanied by satellite cell activation. These cells fuse with existing muscle fibers to support muscle growth [21,22]. Without the activation of satellite cells, postnatal muscle growth and regeneration is severely inhibited. In muscle, it is known that the expression of *Ezh2*, *Mdk*, *Mest*, *Peg3*, and *Plagl1* is increased during regeneration [23-26]. In contrast, *Mycn* and *Gpc3* have not been characterized in muscle. *Mycn*, however, increases proliferation of neural progenitor cells while *Gpc3* is upregulated in the early and middle stages of liver regeneration [27,28]. Because 5 of 7 of these genes are known to be upregulated with muscle regeneration, it is possible that they are involved in muscle growth by increasing activation, proliferation, or differentiation of satellite cells. With age, the expression of all of these genes is expected to decline in both organs and muscle because the growth regulating functions of these genes would be less necessary once growth has ceased.

The objectives of this study were to 1) determine if these 7 genes were downregulated during postnatal growth of skeletal muscle, 2) determine if expression differed between male and female mice during this time period and 3) determine if expression was altered by the absence of myostatin. Myostatin null mice experience increased muscle growth due to both increased hyperplasia and hypertrophy [29] and exhibit increased satellite cell activity [30]. Furthermore, myostatin expression is decreased with increasing age in rats [31], similar to the expression patterns of genes in this study. Therefore, use of myostatin null mice as a model for altered growth to determine the influence of these genes is warranted. In general, we confirm that this set of genes is downregulated with age in skeletal muscle of mice with minimal differences between males and females. Expression of *Ezh2* was increased at d 70 and expression of *Mdk* was increased at d 21 in myostatin null mice compared with wild type, but no other genotype effects were present. These results suggest that this set of genes may globally regulate the cessation of growth in skeletal muscles as well as organs, but likely do not contribute to differences in muscularity of myostatin null mice.

## Methods

All procedures were approved by the University of Illinois Institutional Animal Care and Use Committee prior to beginning the experiment.

### Animal procedures and sample collection

Mice from an internal C57BL/6 J colony were allowed ad libitum access to pelleted rodent chow (Teklad F6 rodent diet, Harlan, Indianapolis, IN, USA) and tap water. Room temperature was maintained at 22°C with a 12 hour light/dark cycle. Male and female mice heterozygous for the myostatin null mutation were bred in monogamous pairs and litters were weaned at 21 days of age. Weaned mice were housed individually and genotyped with PCR based genotyping [29]. At 0 (birth), 21, and 42 and 70 days of age, 5 mice for each genotype by sex combination were euthanized by CO_2_ asphyxiation and decapitation. Only 4 male wild type mice were collected at d 70. Pregnant dams were also euthanized by CO_2_ asphyxiation and decapitation 14 days after breeding (d -7) and 5 fetuses collected to represent each genotype by sex combination. At d -7, the entire mouse body was collected. At d 0, muscles from both hindlimbs were combined. At all other time points, biceps femoris muscles were weighed and collected. Tissues were stored at -80°C until analysis.

### RNA Extraction and cDNA synthesis

Total RNA was extracted using TRI-Reagent (Sigma-Aldrich, St. Louis, MO, USA) and BCP phase separation reagent (Molecular Research Center, Inc., Cincinnati, OH, USA) according to the manufacturers’ protocols. RNA purity and concentration were measured using a NanoDrop 2000c spectrophotometer (Thermo Scientific, Wilmington, DE, USA). For cDNA synthesis, RNA was diluted to 500 ng/μl using RNase-free water and 1 μg of RNA was reversed transcribed using the qScript cDNA Super Mix (Quanta Biosciences, Inc, Gaithersburg, MD).

### Quantitative real-time PCR

Validation experiments were conducted according to the Applied Biosystems quantitative PCR manual (Applied Biosystems, 2004) to determine the appropriate reference gene (Rn18S) and cDNA dilution. Eighteen μl of a 1:100 dilution of cDNA, 2 μl of primer/probe, and 20 μl of PerfeCTa® qPCR FastMix Reaction Mixes (Quanta BioSciences, Inc, USA) were mixed together and plated in 18 μl duplicates into a 96-well plate per reaction. Expression of Ezh2, Gpc3, Mdk, Mest, Mycn, Peg3, and Plagl1 (Table 1) was determined by quantitative real-time PCR using a StepOnePlus Real-Time PCR System thermal cycler (Life Technologies, Grand Island, NY, USA). The thermal cycler performed an initial denaturation at 95°C for 20 seconds followed by 40 cycles of 1 second at 95°C and 20 seconds at 60°C. Expression was normalized in two separate ways. First, to compare the expression of genes with increasing age, expression was normalized to *Rn18s* (ΔCt) and compared to the average of 70-day-old male wild type expression (ΔΔCt). To better visualize the influence of sex and genotype on expression, Ct values were normalized to *Rn18s* (ΔCt) and then compared within the fetal and d 0 time points to the average male wild type average of each time point (ΔΔCt). For 21, 42 and 70 d data, Ct values were normalized to *Rn18s* (ΔCt) and then compared to the average male wild type at 21 d of age (ΔΔCt). Values were expressed as a fold change (2^-ΔΔCt^ = fold change) for statistical analysis.

**Table 1:** ABI primer/probe information

**Gene symbol**	**Gene name**	**Gene bank reference sequence**	**ABI assay ID**
*Ezh2*	Enhancer of zeste homolog 2	NM_007971.2 NM_001146689.1	Mm00468464_m1
*Gpc3*	Glypican 3	NM_016697.3	Mm00516722_m1
*Mdk*	Midkine	NM_010784.4	Mm00440279_m1
*Mest*	Mesoderm specific transcript	NM_008590.1	Mm00484993_m1
*Mycn*	V-myc myelocytomatosis viral related oncogene, neuroblastoma derived	NM_008709.3	Mm00476449_m1
*Peg3*	Paternally expressed 3	NM_008817.2	Mm01337379_m1
*Plagl1*	Pleiomorphic adenoma gene-like 1	NM_009538.2	Mm00494250_m1
*Rn18s*	18S ribosomal RNA	NR_003278.1	Mm03928990_g1

### Statistical analysis

Data were analyzed using the Proc Mixed procedure in SAS version 9.2 (SAS Institute Inc., Cary, NC, USA). The model included the fixed effects of age (when appropriate), sex, and all 2- and 3-way interactions. Data are presented as least squares means ± 95% confidence intervals.

## Results and discussion

### Body and muscle weights

**Figure 1:**
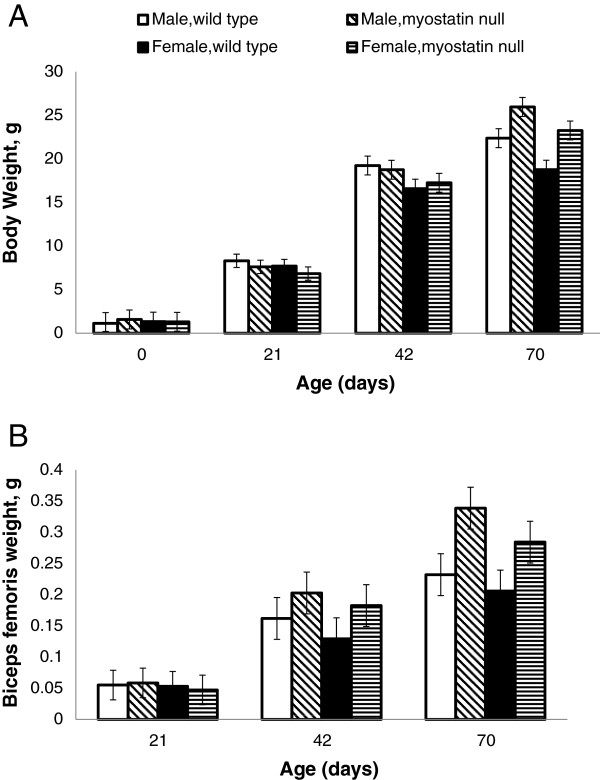
**Body and tissue weights.** Body weight **(A)** and muscle weight **(B)** of male wild type (open bars), female wild type (black bars), male myostatin null (diagonal hatched bars) and female myostatin null mice (horizontal hatched bars). Plotted values are least squares means displayed with 95% confidence interval bars.

Body and muscle weights during postnatal growth were compared between sexes and genotypes to illustrate differences in growth patterns. At day 0 and 21, body weights were similar among all genotype and sex combinations. Mice became heavier with age, and at d 70, myostatin null mice were heavier than wild type mice within sex while males were heavier than females within genotype (Figure 1A). Bicep femoris weights were similar among all treatment groups at d 21 and 42, but myostatin null muscles were heavier than wild type at d 70 (Figure 1B). These results are similar to previous reports of body and muscle weights of myostatin null mice [29,32].

### Expression of growth regulating genes in muscle during growth

**Figure 2:**
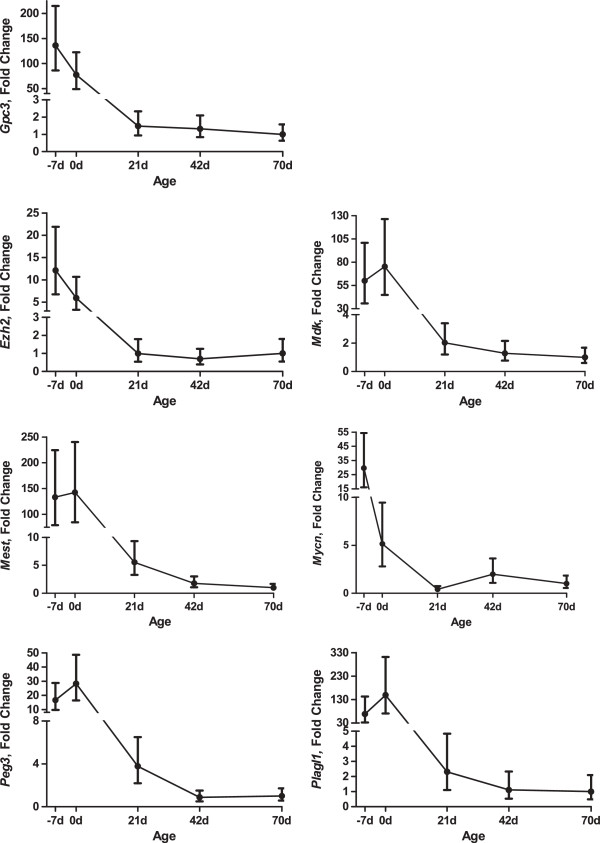
**Expression of growth regulating genes in muscle with age.** Plotted values are least squares means displayed with 95% confidence interval bars. Fold change is compared to the average wild type male 70-day-old expression.

To determine the expression of these genes during growth, the expression of all genes in wild type male mice was analyzed in comparison to d 70 expression (Figure 2). All genes were affected (*P* < 0.01) by age with an overall decline of expression with increasing age. For all genes except *Mycn*, expression was not different between d -7 and d 0; *Mycn* expression was reduced at d 0 compared with d -7. In all genes, expression was reduced (*P* < 0.05) at d 21, d 42 and d 70 compared with d -7 and d 0. Expression of *Ezh2*, *Gpc3*, *Mdk*, and *Plagl1* did not decline further after d 21. However, the expression of *Mest*, and *Peg3* was reduced (*P* < 0.05) at d 42 and 70 compared with d 21, with expression at the later time points being similar. For *Mycn*, expression was reduced (*P* < 0.05) at d 21 compared with d 42 with expression at d 70 being intermediate and similar to either time point. Therefore, the results of previous experiments detailing the expression of these genes in various organs [1,2] are confirmed in skeletal muscle. Expression of these genes declined with age in wild type male mice.

### Expression differences between sexes and genotypes

Expression of all 7 genes was unaffected by genotype or sex at d -7 (Table 2). Similarly, at birth, expression of *Ezh2*, *Mdk*, *Mycn*, and *Plagl1* were unaffected by sex or genotype (Table 3). Furthermore, expression of all genes was unaffected by genotype. Expression of *Gpc3*, *Mest* and *Peg3* were increased in female mice compared with male mice. There were no interactions (P < 0.05) of sex and genotype for expression at d -7 or birth.

**Table 2:** **Effect of sex and genotype on gene expression day -7 (14 d after mating**^**a**^**)**

					**P-value**		
**Gene**	**Male**	**Female**	**Wild type**	**Myostatin null**	**Sex**	**Gen**^**b**^	**Int**^**b**^
*Ezh2*	0.988	1.249	1.273	0.969	0.62	0.56	0.60
*Gpc3*	1.515	1.073	1.158	1.403	0.34	0.60	0.09
*Mdk*	1.038	0.927	0.879	1.094	0.70	0.47	0.63
*Mest*	1.038	0.904	1.000	0.938	0.58	0.80	0.58
*Mycn*	1.155	0.939	1.007	1.077	0.62	0.87	0.59
*Peg3*	0.931	0.849	1.123	0.704	0.83	0.28	0.45
*Plagl1*	1.327	0.995	1.095	1.205	0.58	0.85	0.37

^a^Values are least squares means of fold changes. Fold change was compared to the average wild type male expression.

^b^Gen = Genotype, Int = Interaction of Sex and Genotype.

**Table 3:** **Effect of sex and genotype on gene expression birth (d 0)**^**a**^

					**P-value**		
**Gene**	**Male**	**Female**	**Wild type**	**Myostatin null**	**Sex**	**Gen**^**b**^	**Int**^**b**^
*Ezh2*	0.647	0.924	0.649	0.916	0.95	0.92	0.17
*Gpc3*	0.853	1.253	1.114	0.960	<0.01	0.21	0.16
*Mdk*	0.733	0.763	0.848	0.659	0.89	0.39	0.22
*Mest*	1.057	1.503	1.263	1.257	0.01	0.97	0.31
*Mycn*	0.908	1.051	0.935	1.020	0.55	0.72	0.26
*Peg3*	0.805	1.076	1.018	0.851	0.03	0.17	0.06
*Plagl1*	1.135	1.439	1.211	1.348	0.21	0.57	0.44

^a^Values are least squares means of fold changes. Fold change was compared to the average wild type male expression.

^b^Gen = Genotype, Int = Interaction of Sex and Genotype.

**Figure 3:**
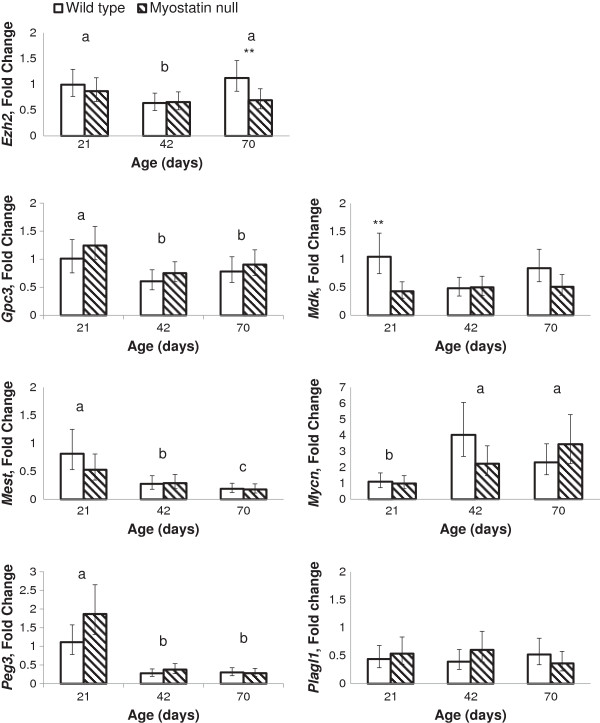
**Comparison of expression of growth regulating genes in skeletal muscle between genotypes at postnatal days 21, 42 and 70.** Plotted values are least squares means displayed with 95% confidence interval bars of wild type (open bars) and myostatin null (hatched bars) mice. Fold change is compared to the average wild type male 70-day-old expression. Days lacking a common superscript (a,b,c) are different (*P* < 0.05). **indicates that means within day are different (*P* ≤ 0.01) between genotypes.

Expression data from d 21, 42 and 70 were compared with the average wild type male expression at d 21 to calculate fold changes. These data represent a consistent muscle group as opposed to the previous data that combine biceps femoris with whole hind limb or fetus (Figure 3). Expression of *Mdk* and *Plagl1* did not decline with age from d 21 to 70. Expression of *Ezh2*, *Gpc3*, and *Peg3* were all increased (P < 0.05) at d 21 compared with d 42 and 70, which were similar to each other. Expression of *Mest* was increased (P < 0.05) at d 21 compared with d 42 and at d 42 compared with d 70. Expression of *Mycn* actually increased with age as expression was increased (P < 0.05) at d 42 and 70 compared with d 21.

Expression of all genes was unaffected by sex. Furthermore, with the exception of *Mdk*, expression was also unaffected by genotype and interactions between genotype with age or sex with age. Expression of *Mdk* was increased (*P* ≤ 0.01) at d 21 in myostatin null mice compared with wild type, but was similar between genotypes at d 42 or 70 (Figure 3). Expression of *Mdk* was also increased (*P* ≤ 0.01) at d 70 in female mice compared with males, but was similar between sexes at d 21 and 42 (data not shown). Expression of *Ezh2* at d 70 was also increased (*P* ≤ 0.01) in wild type mice compared with myostatin null. Therefore, despite differences in muscle weights at d 70 between wild type and myostatin null mice, only the expression of two genes differed at any time point investigated and in both cases, gene expression was increased in wild type, not the more heavily muscled myostatin null mice. Therefore, these data suggest that increases in satellite cell activity and hypertrophy noted in myostatin null mice is not likely due to increased activity in these growth promoting genes.

Nevertheless, we have confirmed that this suite of genes is downregulated with age in skeletal muscle similar to other organs. *Ezh2*, *Mdk*, *Mest*, *Peg3*, and *Plagl1* are known to be involved in muscle regeneration and all of the genes are overly expressed in rhabdomyosarcoma, a skeletal muscle cancer [4-8,23,24,26,33]. It is possible that these genes aid in postnatal muscle growth by activation, proliferation, or differentiation of satellite cells. *Ezh2* has an established role in satellite cell activation [24]; it can be speculated that *Mdk*, *Mest*, *Peg3*, and *Plagl1* might have similar functions because they are also involved in muscle regeneration [23,26]. *Mycn* has not been well characterized in muscle, but it promotes the proliferation of neural progenitor cells and inhibits neural differentiation and, therefore, might exhibit similar functions in muscle with regard to satellite cells [27]. Given these functions, it is expected that expression of these genes would decrease with age as there is less activation, proliferation, and differentiation of satellite cells and, thus, less need for the growth regulatory functions of these genes as growth slows. In contrast, *Gpc3* is thought be a negative regulator of growth such that increased expression results in reduced proliferation [28]. *Gpc3* functions in muscle have not been identified; however, *Gpc3* is involved in liver regeneration such that expression peaks during the early and middle stages of the process [28]. This leads to the conclusion that *Gpc3* expression might increase early in postnatal development to negatively regulate growth and proliferation, so overgrowth of organs and muscle does not occur, and decrease as growth slows because proliferation has decreased leading to less need for its regulatory functions.

## Conclusions

In summary, this study examined the expression decline of 7 growth regulating genes over time in the muscle of wild type and heavily muscled myostatin null mice. Overall, expression was increased in all genes at the fetal time point or birth compared with 21 d of age or later. Despite differences in muscle weights between wild type and myostatin null mice, there were not widespread expression differences between genotypes, nor were there differences between sexes. Therefore, differences observed in muscle weights, especially between those of myostatin null and wild type mice, do not seem to result from differential regulation of the growth-regulating genes examined in this study. It should be noted, however, that the expression of these genes may be different in the satellite or stem cell populations of skeletal muscle compared with the skeletal muscle as a whole. Future work to differentiate the role of these genes in satellite or stem cell proliferation, differentiation and fusion with existing muscle fibers may be warranted.

## Competing interests

The authors declare they have no competing interests.

## Authors’ contributions

JCJ carried out the preliminary studies, established the methods, and drafted the manuscript. KAK carried out the study and performed the statistical analysis. ACD conceived the study, and participated in its design and coordination and helped to draft the manuscript. All authors read and approved the final manuscript.
